# Antibacterial Effect of Aluminum Surfaces Untreated and Treated with a Special Anodizing Based on Titanium Oxide Approved for Food Contact

**DOI:** 10.3390/biology9120456

**Published:** 2020-12-10

**Authors:** Alessandro Di Cerbo, Andrea Mescola, Ramona Iseppi, Roberto Canton, Giacomo Rossi, Roberta Stocchi, Anna Rita Loschi, Andrea Alessandrini, Stefano Rea, Carla Sabia

**Affiliations:** 1 School of Biosciences and Veterinary Medicine, University of Camerino, 62024 Matelica, Italy; Giacomo.rossi@unicam.it (G.R.); roberta.stocchi@unicam.it (R.S.); annarita.loschi@unicam.it (A.R.L.); stefano.rea@unicam.it (S.R.); 2 CNR-Nanoscience Institute-S3, 41125 Modena, Italy; andrea.mescola@nano.cnr.it (A.M.); andrea.alessandrini@unimore.it (A.A.); 3 Department of Life Sciences, University of Modena and Reggio Emilia, 41125 Modena, Italy; ramona.iseppi@unimore.it (R.I.); carla.sabia@unimore.it (C.S.); 4 Moma Nanotech Srl, 20861 Brugherio, Italy; canton@nanotech.it; 5 Department of Physics, Informatics e Mathematics, University of Modena and Reggio Emilia, 41125 Modena, Italy

**Keywords:** bacteriostatic/bactericidal activity, food industry, food contact surfaces, titanium oxide, large-scale roughness, sanitizing treatments

## Abstract

**Simple Summary:**

We firstly described the bacteriostatic activity of aluminum surfaces with three different large-scale roughness against both Gram-negative and Gram-positive bacteria, and the bactericidal activity of three different sanitizing treatments performed on each roughness. Then, we described the bactericidal activity of the same surfaces treated with a special anodizing based on titanium oxide regardless of sanitizing agents and roughness.

**Abstract:**

One of the main concerns of the food industry is microbial adhesion to food contact surfaces and consequent contamination. We evaluated the potential bacteriostatic/bactericidal efficacy of aluminum surfaces with different large-scale roughness (0.25, 0.5 and 1 μm) before and after the surface treatment with a special anodizing based on titanium oxide nanotechnology (DURALTI^®^) and after 3 different sanitizing treatments, e.g., UV, alcohol and a natural product named Gold lotion. Four Gram-negative (*Escherichia coli* ATCC 25922, *Salmonella typhimurium* ATCC 1402, *Yersinia enterocolitica* ATCC 9610 and *Pseudomonas aeruginosa* ATCC 27588) and four Gram-positive (*Staphylococcus aureus* ATCC 6538, *Enterococcus faecalis* ATCC 29212, *Bacillus cereus* ATCC 14579 and *Listeria monocytogenes* NCTT 10888) bacteria were screened. As far as concerns aluminum surfaces without nanotechnology surface treatment, an overall bacteriostatic effect was observed for all strains with respect to the initial inoculum that was 10^6^ CFU/mL. Conversely, an overall bactericidal effect was observed both for Gram-negative and -positive bacteria on DURALTI^®^-treated aluminum disks, regardless of roughness and sanitizing treatment. These results are innovative in terms of the great potential of the antibacterial activity of nanotechnologically treated food contact surfaces and their combination with some sanitizing agents that might be exploited in the food industry.

## 1. Introduction

The food production chain faces daily challenges concerning microbial contamination, in particular on inert structural surfaces of food contact materials (FCM) such as foils for wrapping foods, containers for convenience foods, lids for yogurt containers, tanks for wine, juices, oil, milk, baking trays, meat and sausage hooks, machine parts and utensils for milk processing, which are generally made of aluminum and might be able to produce environments suitable for microorganisms’ proliferation [1,2,3,4]. Most of these microorganisms do not adversely affect food quality or safety, whereas others can, although depending upon the present number [5].

One of the main concerns of the food industry is biofilm development on FCM [3,6,7,8], despite that other surfaces in the food production environment such as floors and walls may act as vectors of microbial contamination through air, personnel and cleaning systems, thus contributing to the overall food quality and safety [9,10].

Surface adhesion is metabolically favorable for bacteria since nutrients are more concentrated at an interface, thus favoring waste product release, replication and consequently, an overall contamination, posing serious concerns in terms of increased fluid frictional resistance and corrosion rate [11,12], decreased heat transfer efficiency [12] and service life of industrial devices [13,14,15,16], and eventually health risks [17,18]. Moreover, once bacteria are attached to surfaces, they can persist even after cleaning treatments [19,20,21], thus paving the way for possible further contaminations.

Depending on food-contact surfaces, cleaning methods can be physical (brushing, scraping or turbulent flow) [22], chemical (alkaline or acid detergents) [23] or a combination of both, while disinfection is achieved by means of disinfectant solutions such as iodine, biguanide, quaternary ammonium compounds, peracetic acid and sodium hypochlorite [24]. Moreover, sanitizing treatments can produce surface topographical defects, increasing the number of attachment sites for microorganisms and leading to corrosion [25]. Nevertheless environmental factors (pH, temperature, ionic strength) [7,26], bacterial cell surface structure [27,28,29] and chemical and physical characteristics of food contact surfaces (elemental composition, hydrophobicity, hydration, charge, free energy, roughness and pores’ presence) [26,30,31,32,33] should also be taken into account since they are able to significantly influence and modulate bacterial adhesion and sanitizing outcome.

The most powerful and versatile tool for investigating surface topography at the nanoscale level is the Atomic Force Microscopy (AFM) [29,34,35,36,37,38]; nevertheless, Environmental Scanning Microscopy (ESEM) has also been extensively used to yield microstructure information of surfaces [39]. In this sense, a detailed evaluation of the food contact surface with regard to the chemical composition and repulsive interaction forces that might generate against bacteria, thus preventing their adhesion, could help food contact surface producers in developing new bacteriostatic/bactericidal strategies such as nanotechnological treatments.

It is noteworthy that, beyond the knowledge of the physical, chemical and thermal behavior and hygiene characteristics (sensitivity to fouling, cleanability and inertness) of materials, manufacturers must also be confident with regulations (European Regulation, EC 1935/2004 [40], 2023/2006 [41], and 764/2008 [42]), standards and guidelines to be applied during food processing equipment construction [43].

The aim of this study was to evaluate the potential bacteriostatic/bactericidal efficacy of aluminum surfaces with different large-scale roughness (0.25, 0.5 and 1 μm) before and after the surface treatment with a special anodizing based on titanium oxide nanotechnology [44], approved for food contact, undergone and not undergone three different sanitizing treatments. Moreover, due to the increasing evidence of the potential flavonoids from citrus fruit as natural antimicrobials mainly against Gram-positive bacteria, we also aimed to provide new insights into their possible use as sanitizing agents against Gram-negative bacteria, too.

Thus, four representative Gram-negative bacteria, *Escherichia coli* ATCC (American Type Culture Collection) 25922, *Salmonella typhimurium* ATCC 1402, *Yersinia enterocolitica* ATCC 9610 and *Pseudomonas aeruginosa* ATCC 27588, and four Gram-positive bacteria, *Staphylococcus aureus* ATCC 6538, *Enterococcus faecalis* ATCC 29212, *Bacillus cereus* ATCC 14579 and *Listeria monocytogenes* NCTT (National Collection of Type Cultures) 10888, frequently detected in the food industry and responsible for foodborne disease outbreaks [8,45,46], were screened. Moreover, topographic analyses of treated and untreated aluminum disks were carried out in a small range by AFM and ESEM.

## 2. Materials and Methods

**Figure 1:**
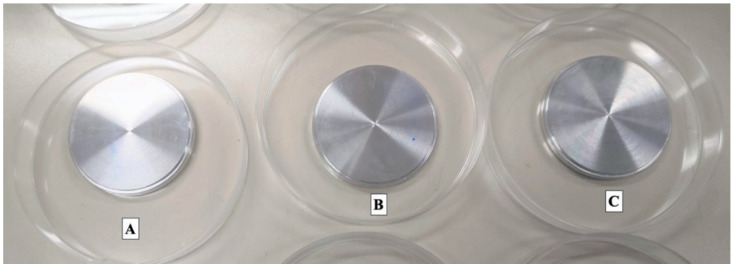
Representative image of aluminum disks with different roughness, (**A**) R0.25 μm, (**B**) R0.5 μm and (**C**) R1 μm.

Two hundred and eighty-eight round-shaped aluminum disks (ANTICORODAL alloy 6082 T6, compliant with European standards, EN 485/573/754/755) with a 5 cm diameter were equally divided into 3 groups by roughness average values and kindly provided SEGAT GIANNI Srl, Gerenzano, Italy (Figure 1). Large-scale roughness (4 mm) was analyzed by profilometer (SURFTEST SJ-210, Mitutoyo Italiana S.r.l., Milano, Italy) resulting in three different roughness average values (R_a_): 0.25 ± 0.02, 0.5 ± 0.03 and 1 ± 0.06 μm, named R0.25, R0.5 and R1, respectively.

**Figure 2:**
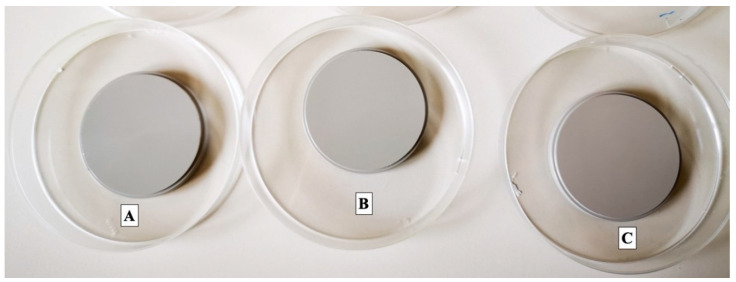
Representative image of aluminum disks with different roughness, (**A**) R0.25, (**B**) R0.5 and (**C**) R1 μm, treated with DURALTI^®^.

After microbiological and microscopic analyses, all disks were covered with a surface treatment named DURALTI^®^ (Gruppo Gaser, Rozzano (MI), Italy) [47], compliant with regulation 1935/2004/CE and National Sanitation Foundation (NSF) standard 51, and therefore suitable for the contact with food products (Figure 2). DURALTI^®^ is a special anodizing obtained from an electrochemical immersion process, which involves the formation of a surface layer of aluminum oxide combined with titanium oxide. The treatment is internal to the surface for about 10 ± 2 μm.

### 2.1. Microbiological Analysis

The stock cultures of *Escherichia coli* ATCC 25922, *Salmonella typhimurium* ATCC 1402, *Yersinia enterocolitica* ATCC 9610, *Pseudomonas aeruginosa* ATCC 27588, *Staphylococcus aureus* ATCC 6538, *Enterococcus faecalis* ATCC 29212, *Bacillus cereus* ATCC 14579 and *Listeria monocytogenes* NCTT 10888 were stored at −20 °C in Tryptic soy broth (TSB) (bioMerieux, Florence, Italy) supplemented with 25% (*v*/*v*) sterile glycerol (bioMerieux, Florence, Italy). Test organisms were first activated by two successive transfers.

### 2.2. Inoculum Preparation

100 μL of the overnight cultures of each bacterium were transferred to 10 mL TSB and incubated at 37 °C with shaking. Absorbance of the cultures were measured at 600 nm after 5 h and the viable cell count at this absorbance was determined by plating onto Tryptic soy agar (TSA). According to the correlation between absorbance and viable cell count, approximately 10^6^ colony forming units (CFU)/mL of each bacterium was inoculated onto aluminum disks.

### 2.3. Sanitizing Procedures and Surface Swabbing

One hundred μL of the inoculum was spread on the surface of aluminum disks with the aid of a sterilized spatula. Thirty-six Petri dishes (12 R0.25, 12 R0.5 and 12 R1) containing one aluminum disk each were tested for each microorganism (total number 288). Twelve disks (4 for each roughness) out of thirty-six underwent one of three different sanitizing procedures for 12 h: UV (UVC, 253 nm), alcohol 70% and Gold lotion (GL, Miyauchi Citrus Research Center, Shigoka-Machi Takasaki Gunma, Japan). The latter is a commercially available natural product made of peels derived from *navel oranges*, *Citrus hassaku*, *Citrus limon*, *Citrus natsudaidai*, *Citrus miyauchi* and *Satsuma*, with a total content of flavonoids equal to 0.45 mg/mL [48]. Alcohol and GL were applied directly on the disk surface with friction in circular movements for 30” by means of a sterile loop. One disk for each roughness was not sanitized and worked as a positive control.

A sterile swabbing was carried out after 12 h by friction of the surface. Next, in sterile conditions, the tip of the swab was placed in a test tube with 1 mL of saline 0.9% and vortexed for one minute. Serial ten-fold dilutions of the obtained re-suspensions were spread onto appropriate agar plates for the viable cell count. The colonies were counted following incubation at 37 °C for 24 h.

### 2.4. Atomic Force Microscopy Analysis

AFM images were acquired with a BioScope I microscope equipped with a Nanoscope IIIA controller (Veeco Metrology, Plainview, NY, USA). The BioScope head was mounted on the top of the samples which have been previously physically attached to the underlying substrate to avoid eventual vibrations potentially harmful for the tip. AFM images were acquired in tapping mode in air, at room temperature, by using triangular doped silicon cantilevers (Veeco, NTESP) with nominal spring constants between 20 and 80 N/m and a resonance frequency around 270 KHz. Processing of AFM images as well as quantification of the roughness were carried out using the free software Gwyddion (version 2.41).

### 2.5. Environmental Scanning Microscopy Analysis

Morphological analysis of the DURALTI^®^ surface-treated aluminum disks was performed by scanning electron microscopy (Nova Nano SEM 450, ThermoFisher Scientific, Monza, Italy) using secondary electrons. Each sample was mounted onto sample stub via double-sided adhesive tape and images were taken at an accelerating voltage of 15 kV.

### 2.6. Statistical Analysis

All the experiments were carried out in triplicate. Data were analyzed using GraphPad Prism 7 software (GraphPad Software, Inc., La Jolla, CA, USA). All data are presented as the means ± standard error of the mean (SEM) and were first checked for normality using the D’Agostino-Pearson normality test. Differences in bacterial growth for each strain at different roughness, both on untreated and DURALTI^®^-treated disks and after different sanitizing methods, were analyzed using a two-way analysis of variance (ANOVA) followed by Tukey’s multiple comparison test. Difference among controls of each strain at different roughness, both on untreated and DURALTI^®^-treated disks, was analyzed using a Kruskal–Wallis test followed by Dunn’s multiple comparison test.

## 3. Results

**Figure 3:**
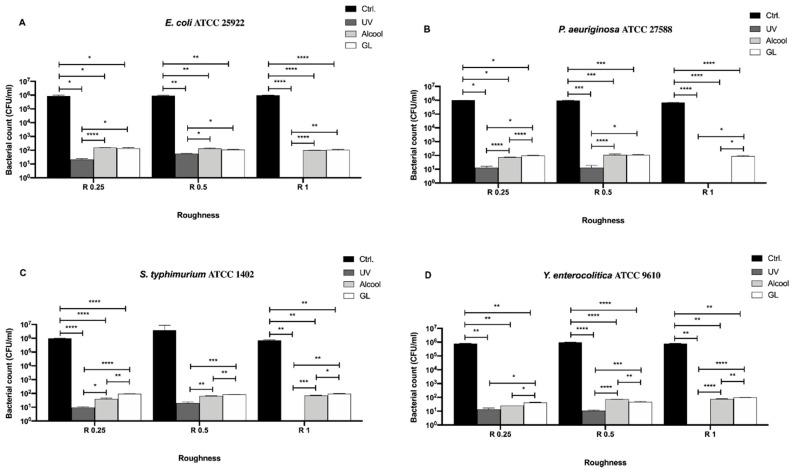
Antibacterial activity of UV, alcohol and GL against Gram-negative bacteria (**A**) *E. coli* ATCC 25922, (**B**) *P. aeruginosa* ATCC 27588, (**C**) *S. typhimurium* ATCC 1402 and (**D**) *Y. enterocolitica* ATCC 9610 at different surface roughness, **** *p* < 0.0001, *** *p* < 0.001, ** *p* < 0.01, * *p* < 0.05.

In Figure 3, differences among the three sanitizing methods (UV, alcohol and GL) and control in different surface roughness (R0.25, R0.5 and R1) against four Gram-negative bacteria (*E. coli* ATCC 25922, *S. typhimurium* ATCC 1402, *Y. enterocolitica* ATCC 9610 and *P. aeruginosa* ATCC 27588) are summarized.

No bacterial count was detectable after UV treatment in R1 for all strains (Figure 3A–D), moreover, also, no detectable count was observed after alcohol treatment in R1 for *P. aeruginosa* (Figure 3B). Regardless of roughness and bacterial strain, an overall significant decrease in bacterial count was observed after each treatment with respect to the control. As for *E. coli* ATCC 25922 (Figure 3A), a significant decrease in bacterial count was observed in R0.25 after UV treatment (21.67 ± 1.66 CFU/mL), with respect to alcohol (156.7 ± 3.33 CFU/mL, *** *p* < 0.001) and GL (140 ± 10.0 CFU/mL, * *p* < 0.05). A similar trend was also observed in R0.5, where the bacterial count after UV treatment was 58.33 ± 1.66 CFU/mL with respect to alcohol (133.3 ± 8.81 CFU/mL, * *p* < 0.05) and GL (110 ± 5.77 CFU/mL, * *p* < 0.05). No significant difference was found between alcohol and GL treatment in R1.

As for *P. aeruginosa* ATCC 27588 (Figure 3B), a significant decrease in bacterial count was observed in R0.25 after UV treatment (13.33 ± 1.67 CFU/mL), with respect to alcohol (73.33 ± 1.66 CFU/mL, **** *p* < 0.0001) and GL (96.67 ± 3.33 CFU/mL, *** *p* < 0.001). Also, alcohol treatment significantly reduced bacterial count with respect to GL (* *p* < 0.05). Conversely, a significant decrease in bacterial count was observed in R0.5 only when comparing UV treatment (13.33 ± 3.33 CFU/mL) with alcohol (110.0 ± 10.0 CFU/mL, * *p* < 0.05) and GL (106.7 ± 6.66 CFU/mL, * *p* < 0.05).

As for *S. typhimurium* ATCC 1402 (Figure 3C), a significant decrease in bacterial count was observed in R0.25 after UV treatment (9.66 ± 0.33 CFU/mL), with respect to alcohol (40.67 ± 2.96 CFU/mL, * *p* < 0.05) and GL (93.33 ± 1.67 CFU/mL, * *p* < 0.05). Further, alcohol treatment significantly reduced bacterial count with respect to GL (*** *p* < 0.001). A similar trend was also observed in R0.5, where the bacterial count after UV treatment was 19.67 ± 2.60 CFU/mL, with respect to alcohol (66.67 ± 1.67 CFU/mL, **** *p* < 0.0001) and GL (83.33 ± 1.66 CFU/mL, *** *p* < 0.001). In addition, alcohol treatment significantly reduced bacterial count with respect to GL (* *p* < 0.05). It is worth pointing out the significant decrease in bacterial count in R1 after alcohol treatment (71.67 ± 1.66 CFU/mL), when compared to GL (96.67 ± 3.33 CFU/mL, * *p* < 0.05).

A significant decrease in *Y. enterocolitica* ATCC 9610 count (Figure 3D) was observed in R0.25 after UV and alcohol treatment (13.33 ± 3.33 and 24.67 ± 0.33 CFU/mL, respectively), when compared to GL (41.67 ± 1.66 CFU/mL, * *p* < 0.05). Almost similar to what was observed for *S. typhimurium* ATCC 1402 in R0.5, a significant decrease in bacterial count was observed after UV treatment (11.0 ± 1.0 CFU/mL) with respect to alcohol (73.33 ± 1.67 CFU/mL, **** *p* < 0.0001) and GL (48.33 ± 1.66, *** *p* < 0.001). Moreover, alcohol treatment also significantly reduced bacterial count with respect to GL (*** *p* < 0.001). As for R1, a significant decrease in bacterial count after alcohol treatment (78.33 ± 1.66 CFU/mL), when compared to GL (99.33 ± 1.67 CFU/mL, ** *p* < 0.01), was also observed.

**Figure 4:**
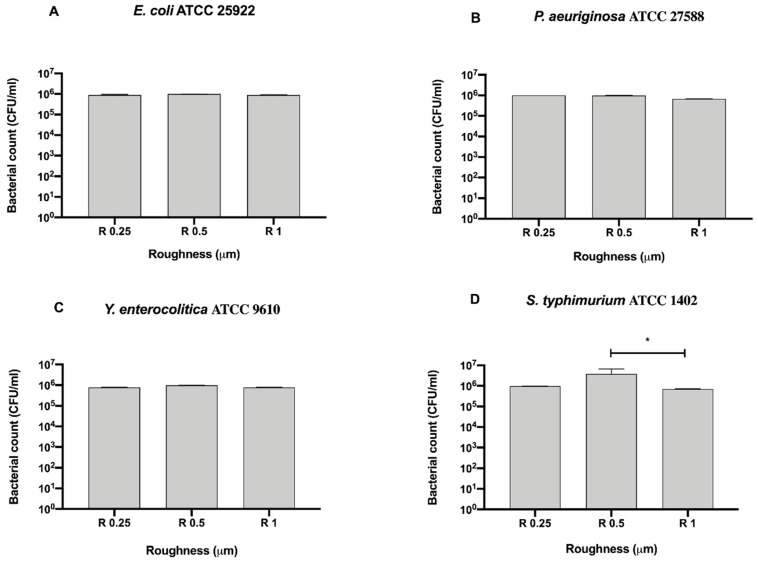
Antibacterial activity of different surface roughness against Gram-negative bacteria (**A**) *E. coli* ATCC 25922, (**B**) *P. aeruginosa* ATCC 27588, (**C**) *S. typhimurium* ATCC 1402 and (**D**) *Y. enterocolitica* ATCC 9610, * *p* < 0.05.

To better address the antibacterial effect possibly exerted by the surface, we further compared the bacterial count of each strain for each surface roughness without a 12 h sanitization with UV, alcohol or GL (Figure 4).

Among the tested roughness, only R1 significantly reduced the count of *S. typhimurium* ATCC 1402 (7 ± 0.28 × 10^5^ CFU/mL), with respect to R0.5 (3.83 ± 2.83 × 10^6^ CFU/mL, * *p* < 0.05) (Figure 4D).

**Figure 5:**
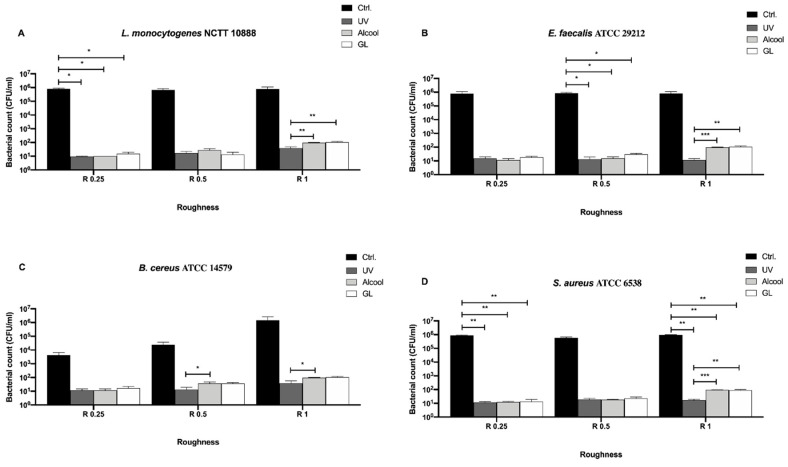
Antibacterial activity of UV, alcohol and GL against Gram-positive bacteria (**A**) *L. monocytogenes* NCTT 10888, (**B**) *E. faecalis* ATCC 29212, (**C**) *B. cereus* ATCC 14579 and (**D**) *S. aureus* ATCC 6538 at different surface roughness, *** *p* < 0.001, ** *p* < 0.01, * *p* < 0.05.

We then also screened the Gram-positive bacteria, *L. monocytogenes* NCTT 10888, *E. faecalis* ATCC 29212, *B. cereus* ATCC 14579 and *S. aureus* ATCC 6538 (Figure 5) and evaluated differences among the three sanitizing methods (UV, alcohol and GL) and control in different surface roughness (R0.25, R0.5 and R1 μm) (Figure 5).

Conversely to Gram-negative bacteria, a complete sanitization of the surface could not be achieved regardless of the roughness and bacterial strain (Figure 5A–D). Despite a visible decrease of bacterial count observed for all strains regardless of treatment and roughness with respect to the control (Figure 5A, B and D), a significant decrease was observed only for *L. monocytogenes* NCTT 10888 in R0.25 (* *p* < 0.05), R0.25 for *E. faecalis* ATCC 29212 (* *p* < 0.05) and R0.25 and R1 for *S. aureus* ATCC 6538 (** *p* < 0.001).

As regards *L. monocytogenes* NCTT 10888 (Figure 5A), a significant decrease in the bacterial count was observed in R1 when using UV (38.33 ± 6.09 CFU/mL) as a sanitizing agent with respect to alcohol (96.67 ± 3.33 CFU/mL, ** *p* < 0.01) and GL (106.7 ± 6.66 CFU/mL, ** *p* < 0.01). A similar trend was observed for *S. aureus* ATCC 6538 (Figure 5D), where a significant decrease in the bacterial count was observed at R1 after UV treatment (16.67 ± 3.33 CFU/mL) as a sanitizing agent with respect to alcohol (93.33 ± 3.33 CFU/mL, *** *p* < 0.001) and GL (90.0 ± 5.77 CFU/mL, ** *p* < 0.01).

As for *E. faecalis* ATCC 29212 (Figure 5B), a significant decrease in the bacterial count was observed in R0.5 and R0.1 when using UV (13.33 ± 7.77 and 11.67 ± 1.66 CFU/mL, respectively) as a sanitizing agent, with respect to GL (30.0 ± 2.88 and 110.0 ± 5.77 CFU/mL, * *p* < 0.01, respectively). When comparing UV and alcohol, a significant decrease was observed only in R1 (11.67 ± 1.66 and 96.67 ± 3.33 CFU/mL, ** *p* < 0.01, respectively). At the same time, a significant decrease was observed in R0.5 when comparing alcohol and GL (15.67 ± 2.33 and 30.0 ± 2.88 CFU/mL, * *p* < 0.01, respectively). Similarly, *B. cereus* growth resulted significantly reduced both in R0.5 and R1 (Figure 5C). In more detail, a significant decrease was observed in R0.5 and R1 after UV treatment (13.33 ± 3.33 and 38.33 ± 10.93 CFU/mL, respectively), with respect to GL (36.67 ± 3.33 and 106.7 ± 6.66 CFU/mL, * *p* < 0.01, respectively). A significant decrease was also observed when comparing alcohol and GL in R1 (96.67 ± 3.33 and 106.7 ± 6.66 CFU/mL, * *p* < 0.01, respectively).

In terms of bacterial count, despite significant differences observed in R1 for all strains and in R0.5 for *E. faecalis* ATCC 29212 and *B. cereus* ATCC 14579 following UV and alcohol treatment, the highest antibacterial activity was reported in R0.25 for all strains. In particular, an overall mean bacterial count of 12.0 ± 0.98, 11.42 ± 0.64 and 15.83 ± 1.35 CFU/mL was achieved using UV, alcohol and GL, respectively.

**Figure 6:**
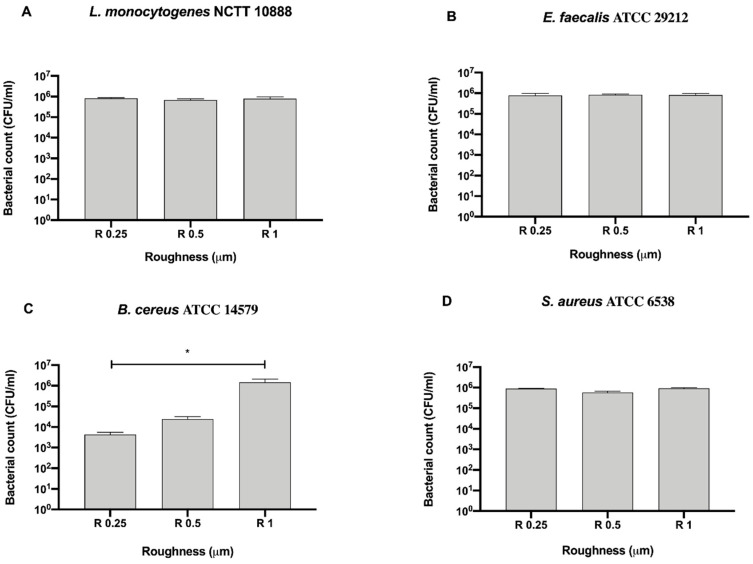
Antibacterial activity of different surface roughness against Gram-positive bacteria (**A**) *L. monocytogenes* NCTT 10888, (**B**) *E. faecalis* ATCC 29212, (**C**) *B. cereus* ATCC 14579 and (**D**) *S. aureus* ATCC 6538, * *p* < 0.05.

As for Gram-negative bacteria, we further compared the bacterial count of each Gram-positive strain for each surface roughness without a 12 h sanitization with UV, alcohol or GL to better address the possible antibacterial effect exerted by the surface (Figure 6).

Among the tested roughness, only R0.25 significantly reduced the count of *B. cereus* ATCC 14579 (4.33 ± 1.20 × 10^3^ CFU/mL) when compared to R1 (1.47 ± 0.62 × 10^6^ CFU/mL, * *p* < 0.05) (Figure 6C).

After microbiological and microscopic analyses, all disks were treated with the DURALTI^®^ anodizing and both analyses were repeated on treated disks.

**Figure 7:**
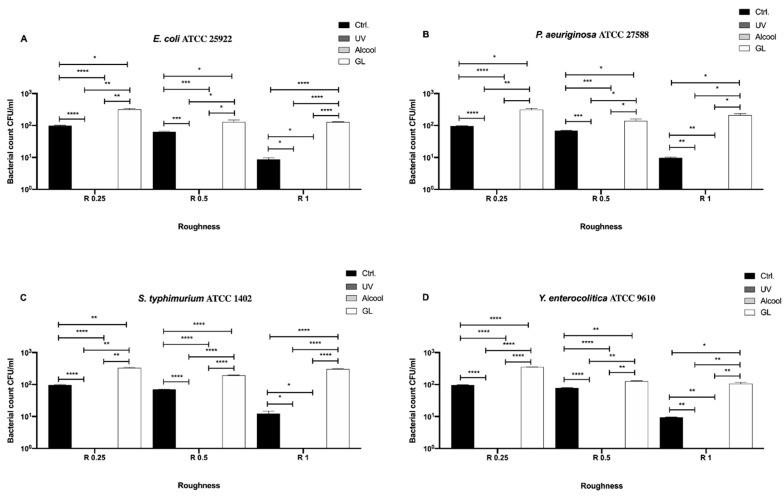
Antibacterial activity of UV, alcohol and GL against Gram-negative bacteria (**A**) *E. coli* ATCC 25922, (**B**) *P. aeruginosa* ATCC 27588, (**C**) *S. typhimurium* ATCC 1402 and (**D**) *Y. enterocolitica* ATCC 9610 at different surface roughness on DURALTI^®^-treated disks, **** *p* < 0.0001, *** *p* < 0.001, ** *p* < 0.01, * *p* < 0.05.

In Figure 7, differences among the three sanitizing methods (UV, alcohol and GL) and control at different surface roughness (R0.25, R0.5 and R1 μm) against the four Gram-negative bacteria are summarized.

Unlike the untreated disks, an overall absence of any bacteria was observed for each strain after treatment with UV and alcohol regardless of the surface roughness (Figure 7A–D). Nevertheless, an overall mean bacterial count reduction could be also visible after treatment with GL (10^2^–10^3^ CFU/mL), which was intriguingly lower than the initial inoculum (10^6^ CFU/mL) regardless of roughness.

Anyway, in both cases, values resulted significantly below the initial inoculum (10^6^ CFU/mL).

**Figure 8:**
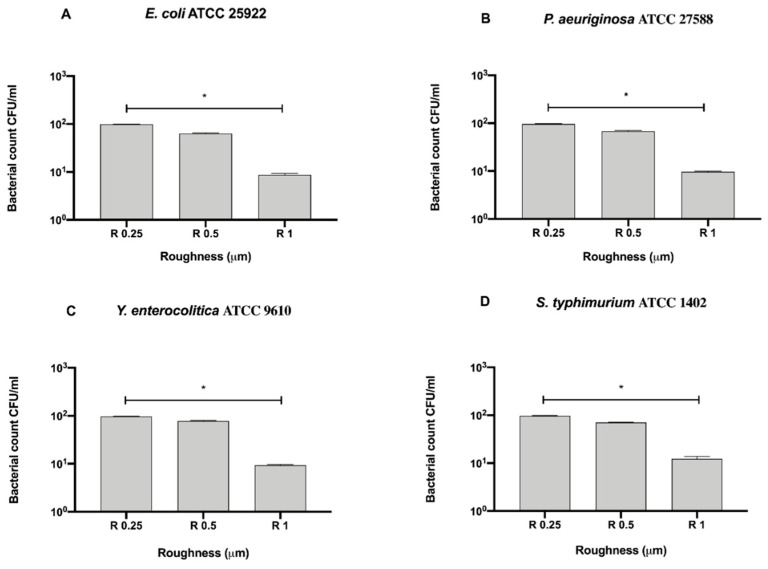
Antibacterial activity of different surface roughness on DURALTI^®^-treated disks against Gram-negative bacteria (**A**) *E. coli* ATCC 25922, (**B**) *P. aeruginosa* ATCC 27588, (**C**) *Y. enterocolitica* ATCC 9610 and (**D**) *S. typhimurium* ATCC 1402, * *p* < 0.05.

To better address the antibacterial effect possibly exerted by the DURALTI^®^ treatment surface, we further compared the bacterial count of each strain for each surface roughness without a 12 h sanitization with UV, alcohol or GL (Figure 8).

Among the tested roughness, only R1 significantly reduced the count of *E. coli* ATCC 25922, *P. aeruginosa* ATCC 27588, *Y. enterocolitica* ATCC 9610 and *S. typhimurium* ATCC 1402 (8.66 ± 0.66, 9.66 ± 0.33, 9.33 ± 1.45 and 12.33 ± 1.45 CFU/mL, respectively), when compared to R0.25 (98.33 ± 1.67, 96.33 ± 1.85, 97.33 ± 1.45 and 97.67 ± 1.45 CFU/mL, * *p* < 0.05, respectively) (Figure 8A–D).

**Figure 9:**
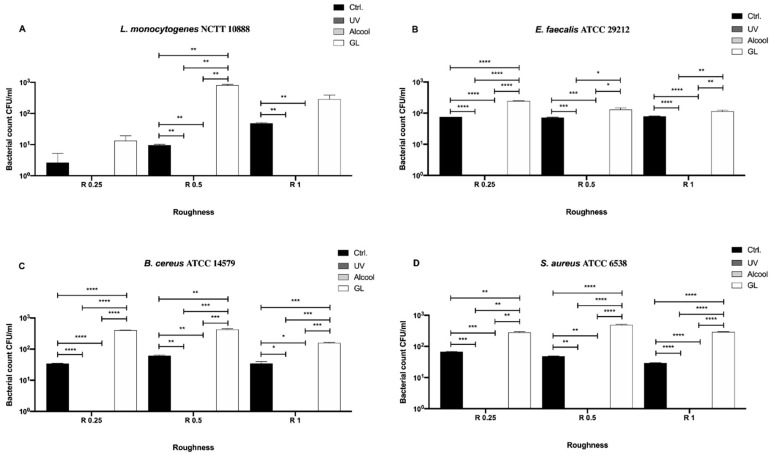
Antibacterial activity of UV, alcohol and GL against Gram-positive bacteria (**A**) *L. monocytogenes* NCTT 10888, (**B**) *E. faecalis* ATCC 29212, (**C**) *B. cereus* ATCC 14579 and (**D**) *S. aureus* ATCC 6538 at different surface roughness on DURALTI^®^-treated disks, **** *p* < 0.0001, *** *p* < 0.001, ** *p* < 0.01, * *p* < 0.05.

As for the untreated disks, we also screened Gram-positive bacteria, *L. monocytogenes* NCTT 10888, *E. faecalis* ATCC 29212, *B. cereus* ATCC 14579 and *S. aureus* ATCC 6538, on the DURALTI^®^ surface treatment (Figure 9).

As noted for Gram-negative bacteria, an overall absence of any microbial count was observed for each strain after treatment with UV and alcohol regardless of the surface roughness (Figure 9A–D). Moreover, as for the control and GL treatment, a similar trend to Gram-negative bacteria was observed. In fact, control resulted intriguingly lower than GL regardless of the surface roughness and bacterial strain. Noteworthy, *L. monocytogenes* NCTT 10888 count resulted almost completely reduced in R0.25 after GL sanitizing treatment (13.33 ± 3.33 CFU/mL), and for the control itself in R0.25 and R0.5 (2.66 ± 1.45 and 9.67 ± 0.33 CFU/mL, respectively), if compared to the initial inoculum (10^6^ CFU/mL) (Figure 9A). Anyway, an overall bacterial count reduction could also be visible after treatment with GL (2.36 ± 0.42 × 10^2^, 4.65 ± 0.73 × 10^2^ and 2.13 ± 0.26 × 10^2^ CFU/mL, respectively), regardless of roughness and if compared with the initial inoculum (10^6^).

**Figure 10:**
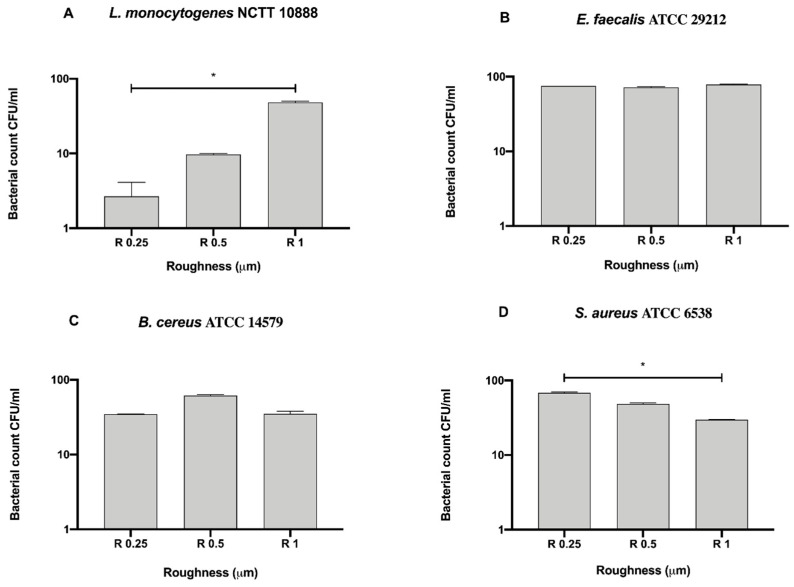
Antibacterial activity of different surface roughness on DURALTI^®^-treated disks against Gram-positive bacteria (**A**) *L. monocytogenes* NCTT 10888, (**B**) *E. faecalis* ATCC 29212, (**C**) *B. cereus* ATCC 14579 and (**D**) *S. aureus* ATCC 6538, * *p* < 0.05.

We further compared the bacterial count of each strain for each surface roughness on DURALTI^®^-treated disks without a 12 h sanitization with UV, alcohol or GL to better address the possible antibacterial effect exerted by the treatment (Figure 10).

*L. monocytogenes* NCTT 10888 resulted significantly inhibited at R0.25 when compared to R1 (2.66 ± 1.45 vs. 48.33 ± 1.67 CFU/mL, * *p* < 0.05, respectively); on the contrary, *S. aureus* ATCC 6538 resulted significantly inhibited at R1 when compared to R0.25 (68.33 ± 1.66 vs. 29.67 ± 0.33 CFU/mL, * *p* < 0.05, respectively) (Figure 10A,D).

**Figure 11:**
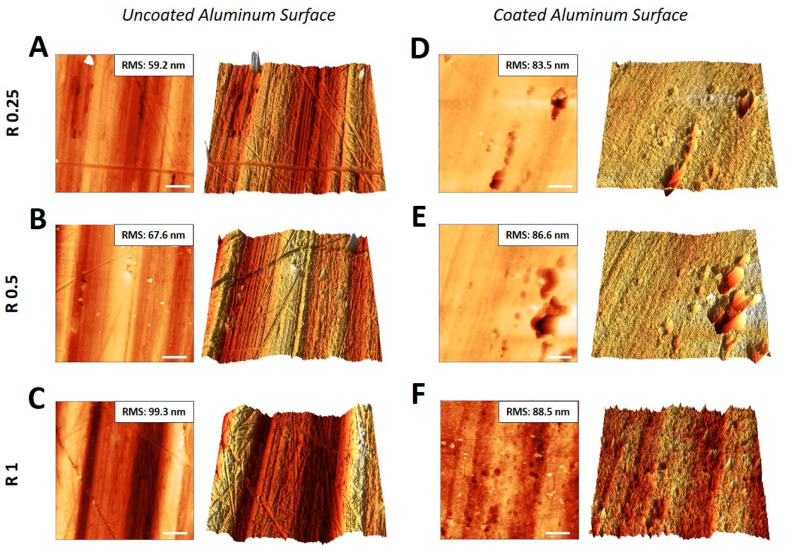
Atomic force microscopy topographic reconstruction of aluminum disk surface before (**A**–**C**) and after (**D**–**F**) DURALTI^®^ surface treatment. Each panel includes planar (**left**) and three-dimensional (3D) reconstruction (**right**). White box in the top right corner of planar reconstruction contains root mean square (RMS) roughness value. Scale bars correspond to 5 μm.

Aluminum surfaces exhibiting different roughness were also examined in a small range by AFM before and after the surface treatment with DURALTI^®^ (Figure 11).

As the technique allows to get insight at a nanometer scale, it is very sensitive to the environmental conditions as well as to the tip size and wear, we decided to analyze several regions of different size at room temperature and to evaluate the root mean square (RMS) roughness over an area of 900 μm^2^.

Images shown in Figure 11 represent topographic reconstruction, both planar and 3D, of aluminum samples before the treatment (Figure 11A–C). Slanting lines over the surface represent typical ripples left by a polishing procedure. The RMS roughness is coherent with the trend observed in larger scale analyses and shows a gradually increasing roughness, from R0.25 to R1. The AFM topographical analysis performed on the DURALTI^®^-treated samples (Figure 11D–F) reveals an almost constant RMS roughness in all the cases, suggesting that the treatment does not affect the initial surface roughness, resulting in a homogeneous structure independently from the initial values of roughness.

**Figure 12:**
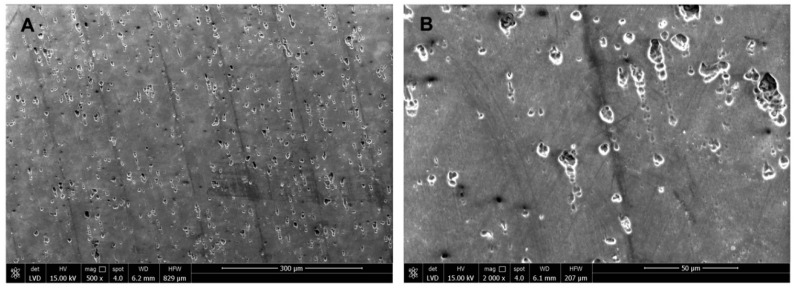
Environmental scanning microscopy morphological analysis on DURALTI^®^-treated aluminum disks surface observed at (**A**) 300 μm and (**B**) 50 μm.

As observed for the AFM, ESEM images acquired on DURALTI^®^-treated aluminum disks confirmed the presence of slanting lines over the surface caused by a polishing procedure observed as well as the presence of diffused and scattered pores and a homogeneous surface treatment (Figure 12).

## 4. Discussion

In the present study, the potential bacteriostatic/bactericidal efficacy of aluminum disks with different surface roughness (R0.25, R0.5 and R1) has been evaluated before and after the surface treatment by means of a nanotechnological anodizing based on titanium oxide (DURALTI^®^) approved for food contact. For such purpose, 8 (4 Gram-negative and 4 Gram-positive) different bacteria have been tested due to their frequent detection in the food industry or their role in foodborne disease outbreaks [8,45,46].

Concerning aluminum surfaces without nanotechnology surface treatment, an overall bacteriostatic effect on the initial inoculum of 10^6^ CFU/mL was observed for all strains. On the other hand, a slight bactericidal effect (reduction by 3 log CFU) was observed on *Bacillus cereus* ATCC 14579 in R0.25 and R0.5. This result is particularly comforting in light of recent evidences that confirmed the ability of *Bacillus cereus* to spread and persist on aluminum surfaces with consequent economic, such as equipment deterioration, and hygienic issues, such as food spoilage [49].

However, it is worth noting that an overall bactericidal effect was then achieved on all bacterial strains regardless of sanitizing treatment and surface roughness. Interestingly, no detectable strains were observed in R1 for all Gram-negative bacteria after 12 h of sanitization with UV and also after 12 h of sanitization with alcohol, 70% for *P. aeruginosa* ATCC 27588. Besides the widely known bactericidal effect of UV [50,51,52] and alcohol [53,54,55], we also confirmed the theory that an increase in surface roughness can negatively affect bacterial adhesion [56,57,58].

Moreover, results suggested a potential use of a natural product, GL, as a sanitizing agent which can be used in place of alkaline detergents, generally responsible for aluminum corrosion [59] and nanoparticle release and accumulation in food, and in turn, in the human body [60]. GL is a natural product rich in flavonoids [48,61], in particular naringin, hesperidin and nobiletin, which already showed strong antibacterial activity against all tested bacteria [62,63,64,65]. This places such a product as a good candidate among sanitizing agents with the additional characteristic of not affecting surface chemical composition and consequent ions/nanoparticle release. Although controversial, we hypothesize that differences, in terms of bacterial count, between GL treatment and control on DURALTI^®^-treated aluminum disks, might be ascribable to chemical interactions occurring at the bacterial strain/GL treatment interface that can slow the antibacterial activity exerted by the surface.

Multiscale topographic analysis conducted by means of AFM allowed us to deeply characterize the surface of aluminum disks, supporting the hypothesis that even at the nanometric scale, surface roughness might be sufficient to elicit a strong response against several bacteria. In fact, as reported by Rizzello et al., morphological, genetic and proteomic changes can occur in adherent *E. coli* as a direct consequence of nanostructured substrates [58].

Contrary to what was reported for untreated aluminum disks, an overall bactericidal effect was observed both for Gram-negative and Gram-positive bacteria in DURALTI^®^-treated aluminum disks regardless of roughness and sanitizing treatment. In particular, no detectable bacteria were visible after UV and alcohol treatment. Interestingly, bacterial count of all controls reached a mean value of 10^2^ CFU/mL, which was similar to that achieved by untreated surfaces after the 12 h sanitization with all three agents. This observation allowed us to speculate a possible bactericidal effect of the anodizing based on titanium oxide, which mainly make up the DURALTI^®^ surface treatment. Such effect became significant once applied all sanitizing agents, thus supposing a synergistic activity characterized by a photocatalytic reaction of both oxides (aluminum and titanium) under UV radiation, as was also demonstrated by different literature reports [66,67,68,69]. The similar bactericidal effects observed after alcohol treatment might be ascribed to a synergistic effect of the friction with the sterile spatula and titanium oxide [66,70] within the DURALTI^®^ coating. In fact, the 6 logarithms reduction observed in our study was in agreement with that observed by Graziano et al. [71].

The topographic analysis of aluminum disks surfaces by AFM has also been performed after exposure to the treatment. The root mean square (RMS) roughness measured did not reveal significant changes when the treatment was performed, confirming that the deposition procedure did not affect the initial roughness and confirming the synergistic bactericidal activity of the treatment under UV radiation. Generally, these findings further confirm the crucial role played by surface topographic features in controlling bacterial adhesion. A proper combination of size, shape and density of such features may seriously affect the bacterial adhesion.

## 5. Conclusions

A bacteriostatic effect was observed for untreated aluminum disk surfaces regardless of roughness, while it became bactericidal after sanitizing treatments’ application. On the contrary, DURALTI^®^ surface treatment induced a bactericidal effect regardless of the surface roughness and sanitizing treatment. These results are innovative in terms of the great potential of the antibacterial activity of nanotechnologically treated surfaces of FCM and their combination with some sanitizing agents that might be exploited in the food industry to reduce the use of corrosive sanitizing agents and allow a longer duration of such nanotechnologically treated materials.

Further studies are needed to better understand the observed phenomena.
